# The Importance of Early Diagnosis and Surgical Intervention in Fractures of the Skull1Read at the semi-annual meeting of the Medical Society of the County of Erie, June 13, 1899.

**Published:** 1899-09

**Authors:** Marshall Clinton

**Affiliations:** Buffalo, N. Y.


BUFFALO MEDICAL JOURNAL.

VOL. XXXIX.—LV. SEPTEMBER, 1899. No. 2.

### Original Communications.

### THE IMPORTANCE OF EARLY DIAGNOSIS AND SURGICAL INTERVENTION IN FRACTURES OF THE SKULL.[1]

By MARSHALL CLINTON, M. D , Buffalo, N. Y.

SEVERAL years ago Bluhm collected a series of over 900 cases, with the intent of demonstrating a lessened mortality in delayed operations following fractures of the skull. These statistics proved that in the immediate operation following injuries the mortality reaches 53 per cent., while in the delayed or later operations, the mortality is only 33 per cent., the other percentage being unknown. In these statistics, however, no reference is made to the ultimate condition of the patients with reference to epilepsy, insanity, and the like, as the result of these injuries; nor does he state what proportion of this 53 per cent. would come under the class of hopeless cases.

We roughly divide fractures of the skull clinically into basilar fractures (including usually the inoperable areas) and fractures of the vault. It is with this latter class we have principally to deal. When fractures of the vault are compound, as extensive or multiple, they are usually recognised readily by simple inspection. When, however, the fracture is a simple one, as a linear fracture, or be it ever so extensive or multiple, the diagnosis is frequently shrouded with more or less doubt.

To the cardinal symptoms, which Phelps lays down as the symptoms of all the fractures of the vault, I should add emphatically from the cases that I have seen—the rapid appearance of a heavily coated, moist tongue, and dry lips in from twelve to twenty-four hours after the time of injury. Too much stress cannot be laid upon

1. Read at the semi-annual meeting of the Medical Society of the County of Erie, June 13, 1899.

the peculiarly doughy feel, usually the accumulation of blood and serum underneath the pericranium, which has been described by Pott. In speaking of the fractures of the bones of the skull it is, of course, recognised that the fractures of these bones, *per se*, are no more serious than those of any other bone of the body; the injury being from the trauma of the brain and adnexa. In all cases of fractured skull, whether they be small in extent or severe, we have a certain amount of hemorrhage from the under surface of the line of fracture, which gives rise undoubtedly to an increase of intracranial pressure. When the hemorrhage is severe, as in the case of a fracture across the middle meningeal artery or its branches, we have in addition to the increase of intracranial pressure, the local pressure of the nerve cells next to the suffused blood, and consequent degeneration from pressure. All cases of fractured skulls presuppose the existence of brain contusion, and these two conditions are so immediately connected with each other that only when no symptom or sign of fracture of the skull exists are we safe in not attempting surgical intervention. In certain instances we see evidences of diffuse brain contusion ; and by brain contusion we mean either actual cell destruction or minute hemorrhages into the cortical brain substances. Injury to skulls, which are resilient in people between the ages of twenty-five and forty, in which the skull's diameters are suddenly changed, allowing of diffused pressure on the cortex without resulting fracture, are particularly difficult of diagnosis, and likewise unsatisfactory to treat.

Case I.—In the case of a sailor who was brought to the Fitch Hospital two years ago, suffering from an injury to the forehead and occiput, no evidence of fracture were found, but the man died five hours later in a condition of intense cerebral irritation—hyperpyrexia —diagnosis of contusion to the cerebellar structures. Post mortem examination showed the diagnosis to be correct and in addition, diffuse contusion to both frontal lobes.

Of course, in this class of cases operative procedure would have been of no avail. In other skulls where the bones are brittle enough to fracture extensively, sparing the underlying brain tissue, we see the reasons for the frequent apparently brilliant results following operations after extensive head injuries.

Case II.—H. M., on September 3, 1897, was struck on the left parietal region by a heavy plank and sustained a compound multiple depressed fracture involving the entire side of his head. On operating upon this patient I found that while the injury to the bone was quite extensive, there was very little, if any, injury to the brain tissue.

Despite the fact that the patient had one-sided paralysis on entrance to the hospital, the rapid relief of this condition by surgical methods before the cells had time to undergo any pressure degenerations resulted in complete recovery and he was discharged on the eleventh day following his injury cured, and since that time has suffered no ill-effects.

Case III.—C. S., aged $3\frac{1}{2}$ years. On the 31st of May, 1897, this little girl was struck on the head by a quoit, and sustained a deeply depressed fracture of the right parietal region. Aside from the respiration of this patient, the symptoms she gave were classically those of contusion described in all the text-books. However, I removed the depressed bone, and the next morning found the child sitting up in bed asking for mamma; cerebral condition relieved. She was discharged cured in a week, and since has had no ill-effects from her injury.

These two cases both illustrate particularly injuries to the bones with slight injury to the brain, which were relieved by prompt operation, in both instances the patients making perfect recoveries.

Case IV.—A. McM. was brought to the hospital eighteen months ago, within twenty minutes of the time of the accident with entire right side of his head crushed. The fracture was simple and presented all cardinal symptoms of cerebral contusion and fracture. On opening the patient's scalp and taking out some particles of bone, I found over the course of the meningeal artery considerable hemorrhage. The brain underlying the fractures was firm; he was pulseless, indicating effusion under the dura. From the extent of the man's injury it was thought that the only chance we had was the most thorough radical kind of relief. This was given him by removing a large portion of the bones from that side of his head, opening the dura in three different places, scraping and washing out the blood until the brain was clean and pulsating, plugging the meningeal vessel, and the wound was dressed. For the first three days the patient showed the symptoms of severe cerebral irritation, but on the fourth day he began to improve, and three weeks later was discharged cured. Since that time he has had no ill-effects from the injury. I have here a fragment of bone, including a part of the orbital plate, which was removed in the effort to reach the sub-dura clot.

Case V.—Frank R., on November 1, 1896, was struck on the side of the head by a picket. His symptoms were those classically of concussion, but as they did not clear up at the end of the third day and were a trifle more severe, it was decided to open up his head and see what the condition actually was. I found hemorrhage from the anterior branches of the middle cerebral artery. The clot was readily removed, the brain apparently regaining its normal position and pulsation. This patient made a rapid recovery and was discharged two weeks later as cured, but twelve months afterward he was seized with his first epileptic fit, and since that time has had them at intervals of three to eight weeks.

I consider this case to illustrate that had this man been trephined at the time of injury he would have had no effects on his brain-cells from continued pressure, therefore no later ill-effects, as shown by his epileptic condition.

Case VI.—Another case of this kind was seen in J. The patient received a severe blow on the right temporal region and it was not deemed wise in the opinion of the attending surgeon to interfere in this case. The patient's condition remained apparently the same from the time of entrance to the seventh day. After a consultation, it was decided to operate upon the man, and examination showed him to have a depressed fracture of the temporal bone, without laceration or marked contusion of the brain tissue. The depressed bone was removed, but the patient made a very tedious recovery, and at the end of the month was transferred to the Buffalo State Hospital as an insane patient. After five months' sojourn there, he was allowed on parole, and eight months later was placed on light duty by the police department. It is safe to assume that in this case had proper examination been made (and by proper examination I mean an incision extensive enough to inspect the area of injury) a recognition of this injury and prompt surgical interference would have resulted in the man's recovery.

The length of time for cell-disintegration or pathological change to take place in the brain following pressure, such as produced by hemorrhage or depressed bone, has not as yet been established. By inference, however, we can see the irreparable harm which may result from improper recognition of cerebral conditions, and what a relatively perfect result can be obtained by the surgeon's use of his best information. As there is no danger in attending a simple incision of the scalp, we have at hand one very simple method of determining, to a great extent, the condition underlying any head injury.

90 N. Pearl Street.

